# Constructing a Learning Curve to Discuss the Medical Treatments and the Effect of Vaccination of COVID-19

**DOI:** 10.3390/healthcare11111591

**Published:** 2023-05-29

**Authors:** Yi-Tui Chen, Emily Chia-Yu Su, Fang Ming Hung, Tomoru Hiramatsu, Tzu-Jen Hung, Chao-Yang Kuo

**Affiliations:** 1 Smart Healthcare Interdisciplinary College, National Taipei University of Nursing and Health Sciences, Taipei 112, Taiwan; 2 Department of Health Care Management, College of Health Technology, National Taipei University of Nursing and Health Sciences, Taipei 112, Taiwan; 3 Department of Education and Research, Taipei City Hospital, Taipei 103, Taiwan; 4 Graduate Institute of Biomedical Informatics, College of Medical Science and Technology, Taipei Medical University, New Taipei City 235, Taiwan; 5 Clinical Big Data Research Center, Taipei Medical University Hospital, Taipei 110, Taiwan; 6 Department of Surgical Intensive Care Unit, Far Eastern Memorial Hospital, New Taipei City 220, Taiwan; 7 School of Policy Studies, Kwansei Gakuin University, Gakuen Uegahara 1, Sanda 669-1330, Japan; 8 Shin Kong Wu Ho-Su Memorial Hospital, Taipei 111, Taiwan

**Keywords:** COVID-19, fatality, learning curves, medical treatments

## Abstract

Acknowledging the extreme risk COVID-19 poses to humans, this paper attempted to analyze and compare case fatality rates, identify the existence of learning curves for COVID-19 medical treatments, and examine the impact of vaccination on fatality rate reduction. Confirmed cases and deaths were extracted from the “Daily Situation Report” provided by the World Health Organization. The results showed that low registration and low viral test rates resulted in low fatality rates, and the learning curve was significant for all countries except China. Treatment for COVID-19 can be improved through repeated experience. Vaccinations in the U.K. and U.S.A. are highly effective in reducing fatality rates, but not in other countries. The positive impact of vaccines may be attributed to higher vaccination rates. In addition to China, this study identified the existence of learning curves for the medical treatment of COVID-19 that can explain the effect of vaccination rates on fatalities.

## 1. Background

As of 20 April 2021, out of a total population of 7.8 billion, more than 3 million cumulative deaths have been reported to the World Health Organization (WHO), corresponding to 38.51 deaths per 10,000 people. In some countries, epidemics have been controlled and stabilized, whereas in others the situation is worsening. By the end of April 2021, the number of newly confirmed cases in some countries remained high, while in some countries, it fell to several hundred. According to the COVID-19 “Weekly Epidemiological Update” published on 27 April 2021 by the WHO [1], the number of new confirmed cases and deaths arising from COVID-19 continue to rise in many countries, with nearly 5.7 million new cases and over 87,000 deaths reported in the preceding week.

Several studies have investigated the causes and effects of COVID-19 fatalities. Shams et al. examined the factors affecting confirmed cases and deaths in different countries and found that people aged > 60 years had higher fatality rates [2]. Additionally, Segovia-Juarez et al. examined the correlation between case fatality rates and altitude and found no significant correlation [3]. However, these studies compared fatality rates across regions or countries and did not reflect fatality trends. Thus, this study attempts to examine the change in fatalities over time by selecting the six countries to be worst hit by COVID-19.

In the early stage of the pandemic of COVID-19, several countries began to develop effective vaccinations. On 30 March 2020, the U.S.A. initiated a program called “Operation Warp Speed” to expedite the development of a COVID-19 vaccine. Moderna and Pfizer achieved preliminary results by employing a messenger RNA (mRNA) vaccine approach. Both companies completed all these Phase III clinical trials by the end of 2020, in which researchers reported an efficacy rate of approximately 95%. In addition to Moderna and Pfizer, 13 vaccines were approved for public use by at least one country: two RNA vaccines (Pfizer-BioN Tech and Moderna), five conventional inactivated vaccines (BBIBP-CorV, Coro-naVac, Covaxin, WIBP-CorV, and CoviVac), four viral vector vaccines (Sputnik V, the Oxford–AstraZeneca, Convidecia, and the Johnson & Johnson), and two protein subunit vaccines (EpiVacCorona and RBD-Dimer) by April 2021 [4].

While various COVID-19 vaccines have been administered worldwide, the supply of vaccines was insufficient to meet demand in the early days following successful vaccine development. At the end of April 2021, based on official reports, more than 1 billion doses had been administered worldwide. China globally ranks highest for COVID-19 vaccinations, with 253.46 million doses administered, followed by the U.S.A. (237.36 million) and India (149.27 million). However, vaccination was not evenly distributed. The total number of vaccination doses in some countries reached more than doses per 100 people, whereas, in the lowest-income countries, it was less than ten doses per 100 people. For example, according to Our World Data, 121.24 and 106.64 doses per 100 people had been administered in Israel and the United Arab Emirates, respectively, by 29 April 2021, whereas in Africa the administration rate was only 1.33 doses per 100 people [5].

Thus, improvement in the treatment techniques through learning-by-doing is still required for medical therapy. The existence of learning curves implies that a repetition of a task or activity may improve performance [6,7]. Learning from past experiences and sharing knowledge within a social system may enhance the adaptability of the social system to external challenges (e.g., acute infection) [8,9].

Furthermore, very few studies have focused on the effect of vaccine administration on COVID-19 fatality based on empirical research in the real world. For example, Herishanu et al. [10] examined the humoral immune responses to the BNT162b2 mRNA COVID-19 vaccine in patients with chronic lymphocytic leukemia to determine the efficacy of the COVID-19 vaccine. Sadarangani et al. [11] employed mathematical models to evaluate the relative effects of COVID-19 vaccines in reducing infection and death based on the assumption that the vaccines have high efficacy and using a fixed value of parameters, such as the probability of infection per contact. Using a group learning process, Peltokorpi and Jaber [12] analyzed performance through the application of knowledge transfer. The results suggest that the S-shaped model (logistic model) provides the best fit for group learning. Several studies used learning theory to predict the relationship between the number of confirmed cases and the social distancing policy such as mask-wearing obtained results that were consistent with the learning theory. For example, Carrignon et al. [13] found that interactions between disease risk and social learning may influence epidemiological dynamics over time. Duffey and Zio [14] applied the theory of learning from errors to predict the recovery time from the outbreak of a pandemic similar to COVID-19 through the use of various measures including medical treatment and non-pharmaceutical interventions. Based on the data provided by many countries, the result of their study found that the infection rate similarly follows the universal learning curve.

This study suggests that learning from past experiences in diagnosing infection may contribute to the development of effective approaches for the treatment of COVID patients and reduce fatality rates if learning effects exist. Past treatment experiences that allow the medical system to attempt various treatments at the initial stage of a pandemic may reduce vulnerability in the future. Accordingly, the paper aimed to (1) examine the existence of the learning effect by employing the concept of “learning-by-doing” from the accumulation of experience and analyze the trend of fatality rates over time and (2) investigate the effect of vaccination on fatality rate reductions. The results may provide valuable information to help authorities decide on suitable response strategies to control the pandemic and reduce the harm of the infection.

## 2. Materials and Methods

### 2.1. Equation and Model Interpretation

Basically, the fatality rate f is calculated as the ratio of total deaths *D* to the total number of confirmed cases *N*, expressed as

(1) f=D/N

As the epidemic has not yet ended in most countries, the hospitalized patients, calculated as the number of confirmed cases, may recover or die later. However, the fatality rate, calculated by the number of deaths divided by the number of confirmed cases in the same period, does not reflect the reality of lethality and treatment performance. Thus, this study developed a calculation formula to estimate the fatality rate, which serves as the performance of medical treatments for COVID-19 patients to reflect the lethality of the disease.

As patients who are discharged alive stay longer in hospitals than those who die during their admission [15], hospitalized patients have a higher probability of surviving than dying. This paper assumes that all confirmed cases, including hospitalized patients, will be alive or dying within the hospital of the length of stay (LOS). Thus, this paper defines the average LOS for COVID-19 patients as a period. The fatality rate *f_t_* in period *t* is defined as the ratio of the number of deaths d in period t divided by the number of confirmed cases *n* in period *t* − 1, expressed as

(2) $$f_{t}=\frac{d_{t}}{n_{t-1}}$$

Considering disease acuity, comorbidities, and other patient characteristics, hospital LOS may vary across hospitals or countries. A retrospective cross-sectional study by Alkundi [15] found that COVID-19 patients without diabetes had hospital LOS of 9.8 days using data provided by the William Harvey Hospital in England between 10 March and 10 May 2020 [15]. Rees et al. [16] found that the median hospital LOS for patients with COVID-19 was 14 days in China and 5 days outside China [15]. The LOS in intensive care units (ICU) is eight days in China and seven days outside China. Garcia et al. found the median LOS was 12 days for critically ill patients with COVID-19 disease in ICUs [17].

ICUs are generally employed to treat critically ill COVID-19 patients and are described as important equipment for saving lives in response to the COVID-19 pandemic. The availability of ICU beds plays a critical role in providing effective treatments for COVID-19 patients. The mortality rate of COVID-19 may rise due to the lack of such devices [18]. Thus, seven days of the median of LOS for COVID-19 patients admitted in ICUs based on the above studies was employed as the period in this study.

In this paper, a theoretical model of learning-by-doing curves is presented to explain the changes in fatalities over time. Arrow first presented a one-factor learning curve, expressed as [19]

(3) $$f_{t}\;=\;\alpha \;N_{t-1}^{\delta }$$

Equation (3) shows that the fatality rate in period *t*, the dependent variable, is a function of the accumulated past experience—which is the total number of confirmed cases treated *N_t_*_−1_ from the first period to period *t* − 1 in medical systems—where *δ* is the learning exponent and α is a scale constant. This model assumes that more experience in treating COVID-19 patients leads to lower fatality rates. As Equation (3) is used to describe the learning curve of medical treatments, reflecting the reduced fatality rate over time, the sign of the learning exponent *δ* should be negative.

In general, all countries may maintain zero deaths in the early periods when the number of confirmed cases is low. The sufficient medical capacity of medical systems may have suppressed death and controlled the spread of the epidemic in earlier periods. However, as time passes, the first death occurs, and the fatality rate increases until it peaks. After peaking, the fatality rate began to decline. To obtain a linear regression model, the logarithm on both sides of Equation (3),

(4) $$lnf_{t}=ln\alpha +\delta \;lnN_{t-1}\;$$

To examine the effect of vaccination on improvement in the epidemic, the vaccination rates variables, which served as an explanatory variable, were incorporated into Equation (4), expressed as

(5) $$lnf_{t}=ln\alpha +\delta \;lnN_{t-1}+\beta \nu_{t-2}$$

where $\nu_{t}$ is the vaccination rate, calculated as the total number of vaccination doses administered per 100 people in the total population, and *β* is the regression coefficient. As the body needs 14 days after vaccination to yield immunity against the virus of COVID-19 and 7 days serve as one period, the vaccination rate calculated in period *t* − 2 is employed to affect the fatality in period t. The statistical analysis of the results was performed using SPSS 19.0 for Windows (IBM SPSS Statistics, Chicago, IL, USA). The results were considered significant at *p* values < 0.01, 0.05, and 0.10.

### 2.2. Criterion of Sample Selection

**Figure 1:**
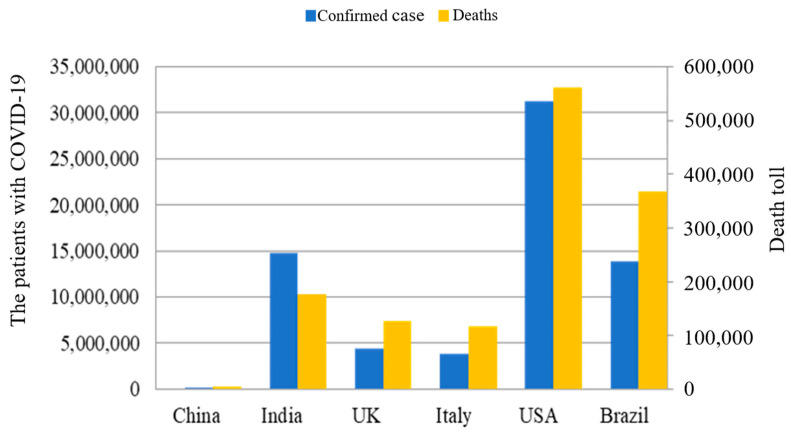
Total confirmed cases and deaths among the six countries.

This study selected six countries, including China, India, Italy, the U.K., the U.S.A., and Brazil, for case studies to examine the learning effect of the treatment for COVID-19 patients. The first case of coronavirus disease was identified in China at the end of 2019. As indicated in Figure 1, by 19 April 2021, the total number of confirmed cases in China had reached 103,273 and the total death toll was 4856.

The U.S.A., India, and Brazil ranked in the top 3 in the highest number of COVID-19 cases and deaths in the world. In the U.S.A., more than 30 million confirmed cases and more than 500,000 deaths were reported. The U.K. and Italy had the two highest COVID-related death tolls among European countries. The high number of deaths and earlier outbreaks of COVID-19 in the U.K. and Italy are valuable for comparing their fatalities with other countries. About 127,000 people had died because of coronavirus in the U.K. Furthermore, the U.K. ranked fourth with respect to total deaths among countries worldwide.

### 2.3. Data Collection

As the first data on vaccination rates were collected from 15 December 2020 (based on the data provided by Our World in Data) [5], the analysis was separated into two stages: (1) the ex-vaccination stage from the date of the first confirmed case to the day before the first dose of vaccination and (2) the post-vaccination stage from the date of the first dose vaccinated to 19 April 2021. In the early stage of the pandemic, the chance of viral genome mutation and the occurrence of newly differentiated variants were lower due to the relatively low number of confirmed cases [20]. However, over time, the evolution of the SARS-CoV-2 genome became more frequent, allowing more variants to emerge. New variants began to spread more easily, rendering some vaccines less effective and causing some patients to develop resistance to therapeutic medicines [21,22]. Several new variants were reported in 2021 and the WHO labeled these “major global variants of concern” as Alpha, Beta, Gamma, Delta, and Omicron at the end of March 2021 [23]. To avoid the effects of new variants, this study discarded data after 19 April 2021. The learning effect only was analyzed in the ex-vaccination stage by regressing Equation (4). In contrast, Equation (5) was employed to analyze both the learning effects and vaccination effects using data on the post-vaccination stage.

The accumulated COVID-19 confirmed cases and deaths were extracted from the website of the “Daily situation report” provided by the WHO [1]. As the date of the first confirmed case and first recorded death for these countries vary greatly, the number of observation points for each country differs. Similarly, the date of the first vaccination for COVID-19 is also different among countries. The number of observation points for each country in the post-vaccination stage differs. Table 1 lists the dates of important events and the accumulated vaccination rates on the date of the first vaccine administered for each country. The first confirmed cases and deaths from COVID-19 occurred in China. Data on total deaths before 2 February 2020 were not available and thus assumed to be zero. On 17 April 2020, a total of 1290 new deaths were reported due to data revision, which was much higher than 0 or 1 death on other days during this period. Thus, the deaths were proportionally added to previous periods. In the U.K., the total number of confirmed cases was updated on 3 July 2020, and nearly 30 thousand cases were excluded. To avoid the emergence of negative confirmed cases during June 2020, the additional confirmed cases were proportionally reduced from the previous periods. According to the database provided by Our World in Data [5], China was the first country to start vaccination for the prevention of COVID-19, and Brazil was the last among the six countries.

## 3. Results

### 3.1. The Trend of Fatality Rates

**Figure 2:**
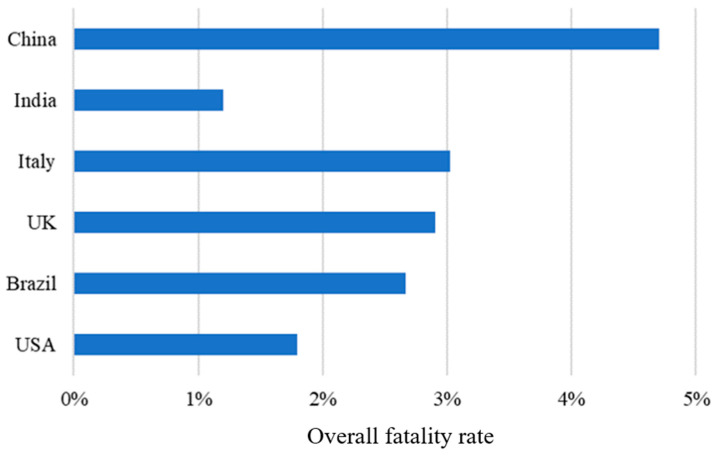
The comparison of overall fatality rates among the six countries.

Figure 2 depicts the overall fatality rate covering the period for each country. Among the six countries, China had the highest fatality rate (4.70%), followed by Italy (3.02%), and India had the lowest fatality rate (1.20%). This section is divided into several subsections. A concise and precise description of the experimental results, their interpretation, and the experimental conclusions are provided.

Traditionally, India is considered a country with poor infrastructure and few medical facilities. The possible cause for low fatality rates in India may be attributed to two reasons: (1) low viral test rates and (2) a lack of effective registration systems. In the early periods, the viral testing rate in India was very low compared with that of other countries [5]. Many deaths due to COVID-19 were recorded as deaths from other causes rather than COVID-19 because no viral tests were performed. Thus, the fatality rate may have been underestimated due to a lack of data. Another reason for the low fatality rate in India may be the low death rates. A substantial delay in the reporting of confirmed cases and deaths may have occurred in India [24]. Only about 70% of deaths caused by COVID-19 were registered in India [25]. Thus, the number of deaths in official records is much lower than the true number, and the overall fatality rate was underestimated in India.

**Figure 3:**
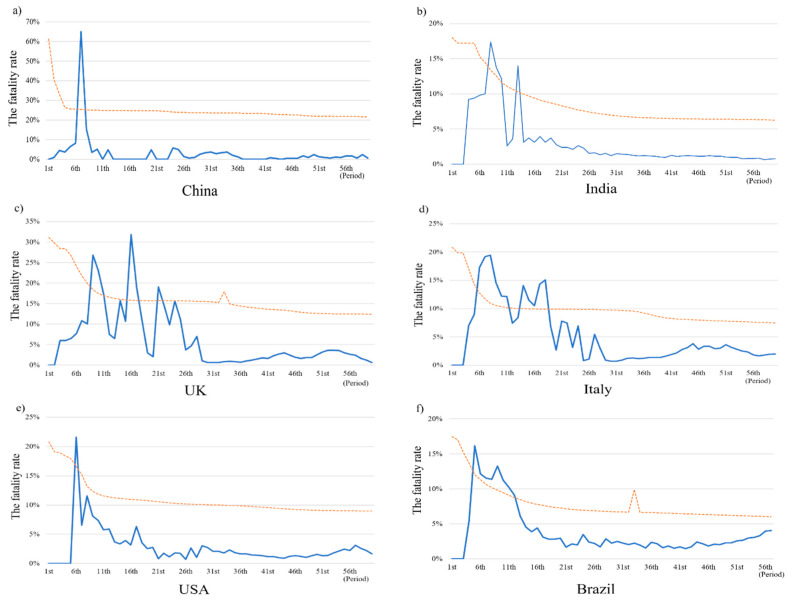
The fatality trend in different countries.

Figure 3 shows the fatality trend over time and the trend line(the yellow dot line) for each country. As can be seen, the fatality rate in China increased sharply to >60% in period 7 and then dropped and fluctuated in the following period. After peaking, it declined sharply and fluctuated. In later periods, the number of deaths fell to 0, 1, or 2, and the fatality rate was reduced to below 1% before the end of 2020 and 1–2% in 2021.

The fatality rate before period 5 (2 March—9 March 2020) in India was zero, as no patients died of COVID-19 and peaked at 17.39% in period 8. Subsequently, the trend fluctuated and maintained a downward trend. The second peak and the third peaks reached 13.97 and 10.59% in periods 13 and 30, respectively. The fatality rate in the U.K. increased very sharply from 0% before period 5 to 26.79% in period 9, and then fluctuated and peaked at 31.81% in period 16. Thereafter, the fatality rate fluctuated and then gradually decreased. The fatality rate trend in Italy very sharply increased from 0% before period 4 and peaked at 19.43% in period 8. After the peak, the fatality rate fluctuated but maintained a downward trend and decreased to 6.13% in period 22. After the second half of 2020, the fatality rate decreased to approximately 1 or 2% but increased to 2–4% at the beginning of 2021. The fatality rate in the U.S.A. increased sharply, peaking at 21.62% in period 6, and then fluctuated and continually decreased. After period 9, the fatality rate remained below 10%. The second peak occurred during period 30 and the rate eventually declined to 1.06% in the last period on 19 April 2021. The zero fatality rates were maintained for only two periods in Brazil. The fatality increased from 5.41% in period 3 to peak at 16.18%. The second peak in the fatality rate occurred in period 8, reaching 13.26%. In periods 11 and 12, the fatality rate fell to below 10%, and then furtherly decreased to below 5%. Similar to that in the USA, the fatality rate decreased in the second half of 2020 and slightly increased in 2021. The fatality rate in the last period was 4.3%, showing a slight increase from 2% in February 2021.

### 3.2. The Effect of Learning in Medical Treatment on Fatality Rates Reduction

Table 2 lists the regression results of Equation (4), which covers the ex-vaccination stage of these six countries. Except for China, the results show that the learning curve was significant for all countries, and the coefficient *δ* for $\ln N_{t-1}$ is significantly negative under a *p*-value less than 5 or 1%. The values of δ are −3.98 × 10^−09^, −6.04 × 10^−08^, −4.83 × 10^−08^, −2.18 × 10^−09^, and −8.24 × 10^−09^ for India, the U.K., Italy, the U.S.A., and Brazil, respectively. The estimated results imply that the fatality rate may continually decline accompanied by an increase in the total number of confirmed cases in the previous period. This means that medical systems can improve the performance of medical treatments through repeated experience.

### 3.3. The Effect of Vaccination on Fatality Rates Reduction

**Figure 4:**
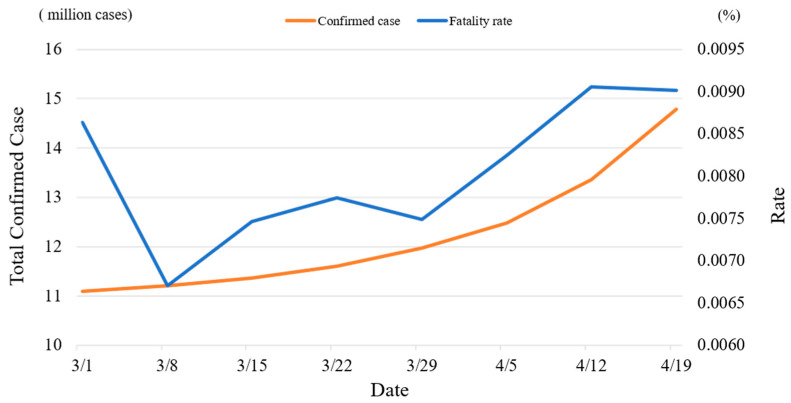
The accumulated confirmed cases and the fatality rate after March 2021 in India.

Table 3 lists the regression results in Equation (5), which covers the post-vaccination stage for these six countries. The result implies that most countries are already experienced and proficient in medical treatment for COVID-19 patients; thus, the learning effect remained significant in the U.K., Italy, the U.S.A., and Brazil. The insignificant learning effect remained the same as that in the ex-vaccination stage in China. However, the learning effect was reversed from the ex-vaccination stage to being insignificant in India in the post-vaccination stage, as shown in Figure 4, which may be attributed to the rapid increase in the number of newly confirmed cases and fatality rates after March 2021. The number of confirmed cases increased from 11.1 million to more than 14 million cases from 1 March to 19 April, with an average growth of 75 thousand cases each day. Especially during the week of 13–19 April 2021, the total confirmed case dramatically increased, with an average of about 204 thousand daily new confirmed cases. Thus, the fatality rate significantly increased. The decrease in the fatality rate on 8 March 2021 may have been caused by the rapid increase in confirmed cases in the previous period. The results also show that vaccination rates are significantly effective in reducing the fatality rate in both the U.K. and the U.S.A. In contrast, the impact of COVID-19 vaccination was not significant in the other four countries.

Table 3 shows that the coefficient δ is positive in the U.S. and the U.K., and the coefficient β is also positive in Italy and Brazil. A positive value of δ implies that the vaccination effect supersedes the learning effect of the medical treatment. Conversely, a positive value of β means that the learning effect remains much higher than the vaccination effect. At the end of April 2021, the vaccination rates in the U.S. and U.K. were 78.4 and 72.2 doses per 100 people, respectively [26]. In comparison, vaccination rates in Italy and Brazil were 33.53 and 21.56 doses per 100 people, respectively. Vaccination rates were much higher in the U.S. and U.K. compared with Italy and Brazil, which explains greater vaccination effects than learning effects in the U.S. and U.K. The regression result in Table 3 fully explains the difference in vaccination effects on the fatality reduction in these four countries.

## 4. Discussion

Except for China, Table 2 shows that the remaining five countries had significant learning effects of medical treatments in response to COVID-19, suggesting that a learning curve may be formed through repeated experience. Previous studies have also emphasized that repeated experiences may improve performance and be positively correlated to case volume and outcomes [27,28]. Kulu et al. [29] examined the impact of surgeon experience on vascular and hemorrhagic complications after kidney transplantation and used case volume to predict the probability of vascular and hemorrhagic complications based on the learning curve [29]. The finding of a significant learning effect in this study is consistent with that of previous studies.

The insignificance of the learning curves for China may be attributed to a lack of experience and knowledge regarding the treatment of COVID-19, given that the epidemic started in China much earlier than in other countries. The initial outbreak of COVID-19 is thought to have occurred at the end of 2019. As the first country to face the newly detected viral threat, no experience or effective medicine was available to help medical systems provide effective medical services for COVID-19 patients. Furthermore, no literature was available as a reference to determine an effective treatment. Research on this newly discovered disease and the development of medicines and vaccines should be timely.

Given this situation, the death toll sharply increased during the earlier periods in China. The first death was reported in period 5 (30 January–6 February 2020). Thereafter, the number of deaths almost doubled in each period until period 7, when it slightly decreased. The peak fatality rate lasted until period 11 (18 March–25 March 2020). The fatality trend shown in Figure 3 indicates that China took more than 80 days to reach its fatality rate.

This implies that a long period of trial-and-errors in China resulted in improved treatment approaches and the treatment procedure gradually became standardized for COVID patients [30]. In the search for effective treatment approaches, China promoted traditional medicines to treat COVID-19, which proved effective in alleviating COVID-19 symptoms and reducing the mortality rate [31]. Based on medical experience, numerous clinical studies focused on effective drugs for the treatment of COVID-19 and proved that some drugs are efficacious in clinical tests [32]. Due to the improvement in medical treatment, instant fatality rates declined and stabilized after period 11.

To develop an effective treatment for COVID-19 patients, China took too much time to reach the peak of the fatality rate and a very short time, 8 and 16 days, to reduce the fatality to below 10 and 5%. Thus, no significant learning effect is found in China.

Currently, several drugs appear to be useful for treating COVID-19. For example, the corticosteroid drug dexamethasone, a low-cost medicine, was shown to decrease the risk of dying in COVID-19 patients through a clinical trial [33]. Remdesivir, an antiviral drug, may reduce the duration of COVID-19 symptoms and has been demonstrated in randomized controlled trials to save lives [34].

The results shown in Table 3 for the post-vaccination stage imply that vaccination rates play a significant role in reducing fatality rates when the vaccine coverage rate reaches a certain level.

**Figure 5:**
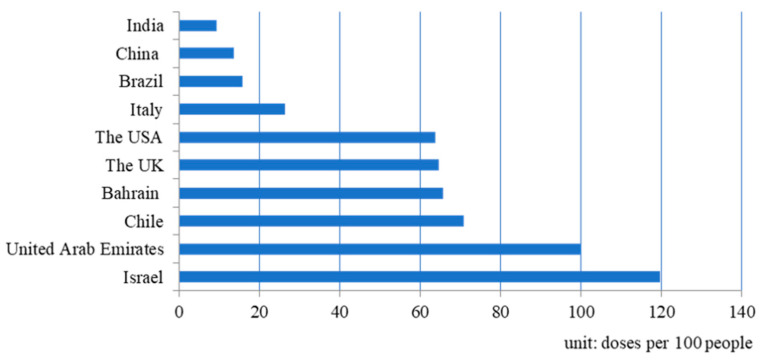
Vaccination rates for the selected countries by 19 April 2021.

The data from the post-vaccination stage implied that vaccination rates positively impact fatality rates when the vaccine coverage rate reaches a certain level. This implies that medical infrastructure plays various roles in the quality of medical treatment. For example, the differences in the capacity of medical resources across countries may affect the vaccination rate and the ability to prevent the spread of disease and develop an effective approach in medical treatments to address a newly evolved disease. In Figure 5, the accumulated vaccination rate of the U.K. ranked the top among the six selected countries, reaching 64.69 doses per 100 people. The U.S.A. was slightly lower, with 63.80 doses per 100 people. India ranked at the bottom, with only 9.39 doses per 100 people. Each administered vaccination was counted as a single dose. As most vaccines require two doses for each person to ensure efficacy, this means that only about 32.35 and 31.9% of the population had been vaccinated in the U.K. and the U.S.A., respectively. The much higher vaccination rates in the U.S.A. and U.K. may explain their effective vaccination. In contrast, the insignificant effect of vaccination in other countries may be attributed to low vaccination rates.

**Figure 6:**
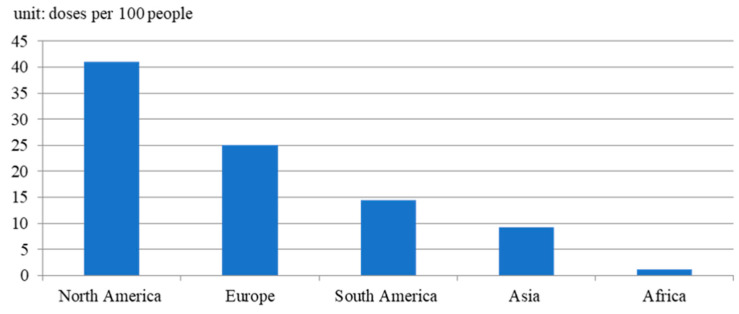
The vaccination rate in each continent by 19 April 2021.

Figure 5 also depicts that four other countries have higher vaccination rates than that of the U.K., including Israel, the United Arab Emirates, Chile, and Bahrain. However, most countries had much lower vaccination rates, even lower than ten doses per 100 people. In Figure 6, in the comparison of vaccination rates between continents, the average vaccination rate of Africa only reached 1.22 doses per 100 people, which is significantly lower than that of other continents. In contrast, North America ranks at the top, with 41.01 doses per 100 people. Considering a vaccination coverage rates threshold of 60–70% of the population (approximately 200 doses per 100 people) may enable a country to gain herd immunity [35]. Herd immunity cannot be reached in the world before the full implementation of vaccination.

By the end of March 2021, 11 vaccines had been developed and globally authorized for clinical use [36]. By the beginning of March 2021, approximately 413 million doses of COVID-19 vaccines had been produced, with projections of 9.5–12 billion doses being manufactured by the end of 2021 [37]. The production capacity of a COVID-19 vaccine is likely to cover a large proportion of the global population. However, the production and distribution of COVID-19 vaccines may be delayed due to the political imposition of export bans for vaccines or manufacturing components [37]. According to the WHO, more than 87% of vaccine doses had been distributed to wealthier countries, whereas low-income countries had only received just 0.2% [38]. Thus, it is difficult to achieve full coverage of vaccination in low-income countries by the end of 2022 [37], and it is still a challenge to attain global herd immunity by the end of 2021.

Furthermore, new variants of COVID-19 that may have more power to transmit and resist vaccines have been identified over the past several weeks. The Centers for Disease Control and Prevention of the U.S. classifies three categories of the SARS-CoV-2 variants being monitored: variant of interest (VOI), variant of concern (VOC), and variant of high consequence (VOHC). Five VOCs are identified in the USA. A new variant with two mutations was detected in India at the end of March 2021 [39]. Preliminary evidence suggests that this new variant, B.1.617, is more contagious [40]. Although most vaccine scientists claimed that their inoculation is still resistant to the viral strain, the risk of infection remains very high. As the pandemic does not seem easy to stop without attaining herd immunity, wearing masks, social distancing measures, and the lockdown policies restricting movement remain effective in slowing the transmission of COVID-19. If the prevention measures adopted by governments worldwide can keep infection levels low and vaccination rates can be accelerated, the transmission of COVID-19 may be significantly reduced in the near future.

### Limitations

Finally, this study has several limitations. First, the observation point ended on 31 July 2020, when the epidemic of COVID-19 in some countries was still maintaining an upward trend. Thus, the fatality rates and learning curves derived in this study for each country are temporarily valid and may change in the future. Second, as many asymptomatic infected individuals exist in society, the actual number of confirmed cases is likely to be much higher than the reported cases. Therefore, the fatality rate may have been overestimated. In contrast, some people may die from COVID-19 but are registered as having other causes on their death certificate owing to a lack of vital tests or being misdiagnosed [41,42,43]. Under such a circumstance, the fatality rate may be underestimated. Thus, the fatality rates calculated in this study may have been either under-estimated or over-estimated. However, the fatality trend should be appropriate, and the significance of the learning curve should remain unchanged, as the events of excess deaths or asymptomatic cases should be maintained at a constant level in each period of the pandemic. Third, this study assumed that death occurred within a LOS of 7 days in the hospital after diagnosis. Thus, the fatality rate and learning curve will change based on a shorter or longer LOS. Fourth, this study neglected the impact of the moderate/mediating variables such as patients’ age, sex, and disease comorbidities.

## 5. Conclusions

This study identified the existence of learning curves for the medical treatment of COVID-19, except in China. The existence of learning effects implies that sharing knowledge about medical treatments for COVID-19 is important for improving the recovery rates of hospitalized patients. Countries that are subjected to later epidemic outbreaks may improve medical treatment by sharing experience and knowledge with countries with earlier outbreaks. Furthermore, the learning curve precisely represents the relationship between the number of accumulated confirmed cases (treated by medical systems) and the fatality rate. This significant learning effect implies that new medical treatments or drugs may be developed to lower fatality rates. However, the increase in the transmission of the epidemic may destroy the reduction in fatality rates caused by learning effects, leading to an increase in total deaths.

This article also explains the effect of vaccination rates on the resulting fatalities in selected countries. The results showed that the fatalities were significantly reduced with the help of higher vaccination rates to increase the proportion of immunized people in society. Considering the insufficient capacity of vaccine production to cover the entire globe and several political interruptions in the equal distribution of vaccines to every corner of the world through export controls, the success of herd immunity to help society to return to normal requires a longer time. Further studies should be conducted to identify the learning curve for medical treatments based on the development of effective medicines or vaccines.

## Figures and Tables

**Table 1:** The date of the first case, death and vaccination, and vaccination rates.

	China	India	UK	Italy	USA	Brazil
First case	2019/12/31	2020/01/30	2020/02/01	2020/01/31	2020/01/23	2020/02/27
First death	2020/02/02	2020/03/14	2020/03/07	2020/02/23	2020/03/03	2020/03/19
First vaccination	2020/12/15	2021/01/16	2021/01/10	2020/12/27	2020/12/20	2021/01/19
Vaccination rate #	0.05	0.01	0.42	0.11	0.46	0.01

# Unit: dose per 100 people.

**Table 2:** The regression result of Equation (4).

	China	India	UK	Italy	USA	Brazil
α	1.24 × 10^−01^	4.50 × 10^−02^ ***	1.04 × 10^−01^ ***	8.76 × 10^−02^ ***	3.79 × 10^−02^ ***	6.79 × 10^−02^ ***
	(8.43 × 10^−02^)	(7.68 × 10^−03^)	(2.55 × 10^−02^)	(1.41 × 10^−02^)	(6.46 × 10^−03^)	(1.27 × 10^−02^)
δ	−9.99 × 10^−07^	−3.98 × 10^−09^ ***	−6.04 × 10^−08^ *	−4.83 × 10^−08^ **	−2.18 × 10^−09^ **	−8.24 × 10^−09^ **
	(9.96 × 10^−07^)	(1.47 × 10^−09^)	(3.29 × 10^−08^)	(2.37 × 10^−08^)	(1.11 × 10^−09^)	(3.37 × 10^−09^)
R^2^	0.1566	0.2952	0.222	0.3034	0.2952	0.2952
Observation number	46	50	47	45	46	44

(): standard errors, *: *p* < 0.10, **: *p* < 0.05, ***: *p* < 0.01.

**Table 3:** The regression result of Equation (5) covers the period of the post-vaccination Stage.

	China	India	UK	Italy	USA	Brazil
α	−8.45 × 10^−03^	1.68 × 10^−04^	−6.61 × 10^−02^ ***	7.42 × 10^−02^ ***	−4.57 × 10^−02^ **	3.73 × 10^−02^ **
	(9.94 × 10^−02^)	(2.38 × 10^−02^)	(1.53 × 10^−02^)	(1.85 × 10^−02^)	1.84 × 10^−02^	(1.27 × 10^−02^)
δ	2.17 × 10^−07^	8.43 × 10^−10^	3.10 × 10^−08^ ***	−1.93 × 10^−08^ **	2.81 × 10^−09^ ***	−2.11 × 10^−09^
	(1.02 × 10^−06^)	(2.25 × 10^−09^)	(4.88 × 10^−09^)	(8.52 × 10^−09^)	8.01 × 10^−10^	(1.48 × 10^−09^)
β	−7.34 × 10^−04^	−3.73 × 10^−04^	−9.66 × 10^−04^ ***	6.77 × 10^−04^	−3.95 × 10^−04^ **	2.03 × 10^−03^
	(8.19 × 10^−04^)	(5.92 × 10^−04^)	(1.34 × 10^−04^)	(5.94 × 10^−04^)	1.31 × 10^−04^	(1.99 × 10^−03^)
R^2^	0.1292	0.1228	0.0945	0.3034	0.462	0.8509
Observation number	17	12	13	15	16	12

(): standard errors, **: *p* < 0.05, ***: *p* < 0.01.

## Data Availability

The datasets generated and/or analyzed during the current study are available in the WHO repository, https://covid19.who.int/data (accessed on 25 May 2021).
